# A critical review of “Artificial intelligence in the practice of forensic medicine: a scoping review”

**DOI:** 10.1007/s00414-024-03209-z

**Published:** 2024-04-01

**Authors:** Benjamin Ondruschka, Dragana Seifert, Natalie Rotermund, Anselm Fehnker

**Affiliations:** 1 Institute of Legal Medicine, University Medical Center Hamburg-Eppendorf, Hamburg, Germany; 2 Artificial Intelligence Center Hamburg (ARIC) e.V, Hamburg, Germany

Dear Editor-in-Chief,

We recently read the article “Artificial intelligence in the practice of forensic medicine: A scoping review” [1] published in your esteemed journal with great interest. While the article provides a comprehensive review of the role of artificial intelligence (AI) in forensic medicine, we would like to offer some critical reflections to fuel upcoming research on this emerging topic with technical knowledge and scientific evidence. Up to now, no single AI method is used globally as standard diagnostic procedure in forensic pathology and thus, has made its way from research to daily practice. However, all of us are aware that this moment will come and perhaps is sooner realized than later.

Firstly, we absolutely agree with the authors’ stance that the formulation of recommendations and guidelines for good practice by national and international scientific societies is a vital step towards achieving usefulness and compliance. Even further, it is of high importance right now to demand legal guidance to develop and implement AI applications that will meet regulatory and legal requirements especially in case of forensic pathology questions. Contributions on this topic from the community and the legal sciences are therefore highly desirable. However, regarding some aspects, we want to provide suggestions for added value, such as the definition of AI and non-AI methods, a combined market analysis and the use of generative AI in the field of forensic medicine.

In the fast-evolving field of AI, a precise definition is basis to solid assessments and required for all conclusions drawn. The definition of ‘AI being a model integrated in a computer program or a part of a computer program that performs a specific task’ is a very wide definition, potentially leading to misunderstandings. Especially as later in the paper, non-AI methods are compared to AI methods. Here, it remains unclear to what extent the non-AI methods do not fulfill the previous definition of AI. A more precise differentiation and an explanation of what is covered by the term non-AI methods would be helpful to better understand the results of the comparison.

While the review gives an overview and describes state-of-the-art AI applications used by forensic pathologists and their levels of integration in medicolegal practices, based on research papers, the information content from other sources should not be underrated and at least be discussed. For example, it would certainly be interesting to explore whether a combined market analysis comes to a similar conclusion compared to the approach taken, or if commercial applications, also considering AI modules embedded in software where the forensic pathologist is not the final user, are already in regular use. One forensic example used throughout the world is the customized ‘death time estimation’ software using the nomogram method by Henssge, although not applying on AI.

One area of AI that is not mentioned in the overview but is developing rapidly, especially in light of the introduction of ChatGPT, is the area of generative AI. It would therefore be important to investigate whether there are potential applications of generative AI in forensic medicine and, if so, whether or not they are already being used. However, generative AI could also be problematic in case of mis-use for diagnostic purposes when expert opinions may include information gathered through GPT but are not reflected thoroughly by the forensic expert.

We want to emphasize that the given review makes a laudable contribution, especially at the present time, as it provides a basis for opening the discussion on the role of AI in forensic medicine and sets the stage for further research and debate in this area next to further papers on this hot topic published almost simultaneously [2]. It is now, where it is necessary to seize the opportunities offered by AI but at the same time maintain the high standards of the field.
